# *Enterococcus hirae* LcpA (Psr), a new peptidoglycan-binding protein localized at the division site

**DOI:** 10.1186/s12866-016-0844-y

**Published:** 2016-10-12

**Authors:** Maxime Maréchal, Ana Amoroso, Cécile Morlot, Thierry Vernet, Jacques Coyette, Bernard Joris

**Affiliations:** 1 Physiologie et génétique bactérienne, Centre d’Ingénierie des Protéines, Université de Liège, Institut de Chimie, Liège, B-4000 Belgium; 2 University Grenoble Alpes, IBS, Grenoble, F-38044 France; 3 CNRS, IBS, Grenoble, F-38044 France; 4 CEA, IBS, Grenoble, F-38044 France

**Keywords:** Enterococcus, Cell wall, Peptidoglycan, Bacterial division, LytR-CpsA-Psr

## Abstract

**Background:**

Proteins from the LytR-CpsA-Psr family are found in almost all Gram-positive bacteria. Although LCP proteins have been studied in other pathogens, their functions in enterococci remain uncharacterized. The Psr protein from *Enterococcus hirae*, here renamed LcpA, previously associated with the regulation of the expression of the low-affinity PBP5 and β-lactam resistance, has been characterized.

**Results:**

LcpA protein of *E. hirae* ATCC 9790 has been produced and purified with and without its transmembrane helix. LcpA appears, through different methods, to be localized in the membrane, in agreement with *in silico* predictions. The interaction of LcpA with *E. hirae* cell wall indicates that LcpA binds enterococcal peptidoglycan, regardless of the presence of secondary cell wall polymers. Immunolocalization experiments showed that LcpA and PBP5 are localized at the division site of *E. hirae*.

**Conclusions:**

LcpA belongs to the LytR-CpsA-Psr family. Its topology, localization and binding to peptidoglycan support, together with previous observations on defective mutants, that LcpA plays a role related to the cell wall metabolism, probably acting as a phosphotransferase catalyzing the attachment of cell wall polymers to the peptidoglycan.

**Electronic supplementary material:**

The online version of this article (doi:10.1186/s12866-016-0844-y) contains supplementary material, which is available to authorized users.

## Background

Enterococci are Gram-positive, facultative anaerobic cocci commensal bacteria [1]. These bacteria make up part of the normal human fecal flora but nowadays several enterococcal species emerge among major pathogens in hospital intensive care units. It is believed that their high prevalence in infections is somehow influenced by their resistance to the most commonly used antibiotics, along with the acquisition of virulence factors and their ability to form biofilms. Enterococcal’s growth depends on cell wall (CW) synthesis. The cell wall is composed of three major constituents: peptidoglycan (PG), secondary cell wall polymers (SCWP), including teichoic acids and cell wall polysaccharides directly tethered to peptidoglycan through covalent linkages, and wall associated or wall anchored proteins [2]. PG is a polymer made of adjacent glycan chains consisting of the repeating disaccharide N-acetylmuramic acid-(β1-4)-N-acetylglucosamine cross-linked by peptides [3]. Most of the enzymes that catalyze the last steps of PG synthesis are the Penicillin-Binding Proteins (PBPs). Some of them polymerize the glycan chains of the PG through their transglycosylase activity and reticulate the peptide chains through their transpeptidase activity. Penicillin (included in the β-lactam antibiotic family) binds covalently to these enzymes thereby inhibiting their transpeptidase activity. Enterococci are intrinsically resistant to β-lactams due to the expression of a PBP displaying a low affinity for these antibiotics [4, 5].

*Enterococcus hirae*, a species belonging to the *Enterococcus faecium* species group [6], was often used as a model organism for the study of PBPs. In *E. hirae* and *E. faecium*, the low-affinity PBP is a class B PBP designated as PBP5. In the presence of therapeutic β-lactam concentrations inhibiting all other PBPs, PBP5 keeps its transpeptidase activity, which in association with a monofunctional glycosyltransferase, allows synthesis of PG, as well as a normal growth [7–11]. The *pbp5* gene is located in the chromosome downstream the *psr* (*PBP5* synthesis repressor) and *ftsW* (filamenting temperature sensitive W) genes in both *E. hirae* and *E. faecium* genomes [12].

Among enterococci species, wall teichoic acids (WTA) differ in structure and complexity. The well-conserved WTA biosynthetic operons from *Bacillus* and *Staphylococcus* species appear only partly present in enterococcal species or those functions relating to WTA biosynthesis are encoded within different CW polymers synthetic pathways [13]. *Enterococcus faecalis* and *E. faecium* harbor a *epa* (enterococcal polysaccharide antigen) cluster containing genes that are predicted to encode proteins involved in the synthesis and the export of repeating units of Epa polysaccharide. The Epa polysaccharide structure has not yet been elucidated, but it is composed of glucose, rhamnose, N-acetyl glucosamine, N-acetyl galactosamine, galactose and probably phosphate [14, 15].

The Psr protein from *E. hirae* has been associated with the regulation of the expression of the low-affinity PBP5 and β-lactam resistance [16], based mainly on the fact that PBP5 is overexpressed in the *E. hirae* mutant R40, which harbors an 87-bp deletion that overlaps the start codon of the *psr* gene, thus impairing the translation of the Psr protein. Therefore, Psr was assumed to repress the synthesis of PBP5. Psr has also been involved in the regulation of several additional surface-related properties, including increased sensitivity to lysozyme, increased rate of cellular autolysis and decrease of the rhamnose amount in the cell wall [17–19]. However, the fact that R40 is not an isogenic mutant of the wild type strain [7] implies that in addition to the truncated *psr* gene, other mutations could contribute to PBP5 overproduction, β-lactam resistance and/or cell wall modifications. Indeed several observations support this assumption. It was demonstrated in *E. faecium* that the quantity of *pbp5* transcript was similar in wild type and *psr*-deficient strains [20]. It was also shown that in an *Enterococcus faecalis* JH2-2r clone resistant to β-lactams, overproduction of the low-affinity PBP (PBP4) is not related to a *psr*-like gene [21]. Finally, deletion of the *psr* gene in *E. hirae* ATCC 9790 has no regulatory effect either on the transcription of *pbp5* or on the autolysis of *E. hirae* cells [22]. Therefore, the specific function of Psr in enterococci remains unknown.

The *psr* gene encodes a protein of 293 amino acids belonging to the LytR-CpsA-Psr family (LCP), a group of proteins that possess a conserved 150 amino-acid LCP domain [23, 24]. The LCP proteins are found in almost all Gram-positive bacteria and several organisms contain more than one LCP protein [24].

The phylogenetic distribution of the LCP family in different subgroups might explain the multitude of reported functions for the LCP proteins depending on the bacterial species that is studied [24]. Single, double, and triple deletion mutants for LCP paralogues were studied in *Staphylococcus aureus* [25], *Bacillus subtilis* [26] and *Streptococcus mutans* [27]. LCP-deficient mutants show that at least one functional LCP protein is required for viability [26, 27] and normal growth [25]. Phenotypes of single, double or complemented mutants indicate a certain redundancy in their functions but also specific differences between those LCP proteins [25–27]. Many roles were attributed to LCP proteins, including cell surface properties, autolysin activity, virulence, antibiotic resistance and septum formation. Recent works have shown that this family of proteins is involved in the transfer of SCWP from lipid carriers to the PG in different bacterial species [26, 28–31].

In *Actinomyces oris*, a glycosylation pathway involving a sortase and LCP proteins has been described. It was proposed that LCP and sortase enzymes work on the same protein substrate (AcaC/GspA). The LCP catalyzes the glycosylation of GspA before the sortase covalently attaches GspA to PG for surface display [32, 33].

Intriguingly, six LCP homologues are present in *Bacillus anthracis* genome although this species does not synthesize WTA. These LCP enzymes were shown to promote attachment of CW polysaccharides to PG at discrete locations, linked to the division site [34, 35]. In support of the idea that *B. anthracis* LCPs catalyze the transfer of SCWP, heterologous expression experiments in *S. aureus* showed that *B. anthracis* LCPs are able to attach teichoic acid to *S. aureus* PG.

The CpsA protein in *Streptococcus agalactiae* is involved in capsule expression and CW stability [36]. A septal localization was demonstrated for CpsA in that species [37], like other LCP proteins in *S. pneumoniae* [28]. Another LCP protein from *Mycobacterium marinum*, named CpsA, is involved in cell wall integrity and is required for mycobacterial virulence [38].

The LCP proteins have also been recently proposed to transfer rhamnose CW polymers from the flipped lipid carrier onto the PG, but experimental evidence for such hypothesis is still lacking [39].

The Psr protein of enterococci is poorly studied. In accordance with available studies on proteins belonging to the LCP family, Psr was renamed LcpA (see below). Although the LCP proteins have been studied in other pathogens, their function(s) in enterococci remain uncharacterized. In the present study, we investigated the characteristics of LcpA from *E. hirae* ATCC 9790. Two forms of LcpA were overproduced and purified. The structural organization and the topology of LcpA were studied. Its cellular localization and interaction with PG were investigated.

## Methods

### Bacterial strains, media and growth conditions

The *Escherichia coli* and *E. hirae* strains used in this study are listed in Table 1. *E. coli* DH5α was used to generate and maintain recombinant plasmids in Luria-Bertani medium (Difco) at 37 °C. *E. coli* Rosetta (DE3) pLysS was used to overexpress recombinant protein from *E. hirae. E. coli* Top10 was used to express the fusion LcpA-BlaM. *E. hirae* strains were grown in Brain Heart Infusion broth (Difco) at 37 °C.

**Table 1:** Bacterial strains and plasmids used in this study

Strain or plasmid	Genotype and/or relevant characteristic	Source or reference
Strains		
*E.coli* DH5α	F– Φ80*lac*ZΔM15 Δ (*lac*ZYA-*arg*F) U169 *rec*A1 *end*A1 *hsd*R17 (rK–, mK+) *pho*A *sup*E44 λ– *thi*-1 *gyr*A96 *rel*A1	Invitrogen
*E. coli* Top10	F– *mcr*A Δ (*mrr*-*hsd*RMS-*mcr*BC) Φ80*lac*ZΔM15 Δ*lac*X74 *rec*A1 *ara*D139 Δ (*ara leu*) 7697 *gal*U *gal*K *rps*L (StrR) *end*A1 *nup*G	Invitrogen
*E. coli* Rosetta (DE3) pLysS	F^−^ *ompT hsdS*_B_ (r_B_^−^ m_B_^−^) *gal dcm* (DE3) pLysSRARE (Cam^R^)	Novagen
*E. hirae* ATCC 9790	Wild-type strain (MIC of PenG : 0.6 μg/ml)	American Type Culture Collection
*E. hirae* FS4	ATCC 9790 psr::Km^r^ (substitution)	Sapunaric et al., 2003 [22] (17)
*E. hirae* R40	Pen^r^ mutant derived from ATCC 9790 (MIC of PenG : 60 μg/ml)	Fontana et al., 1983 [7] (5)
Plasmids		
pET-22b	Expression vector, amp^r^	Novagen
pET-22b-*lcpA*	Expression vector carrying *lcpA*, amp^r^	This study
pET-22b-*lcpA*_*30–293*_	Expression vector carrying the truncated form of *lcpA* gene, amp^r^	This study
pCIPblaM	Reporter vector using TEM β-lactamase, Km^r^	Chahbounel et al., 2005 [40] (27)
pDML1677	Reporter vector carrying *lcpA-blaM* in-frame fusion, Km^r^	This study

### Genetic constructions

Plasmids for the production of recombinant LcpA and its LcpA_30–293_ truncated form (TM helix deleted) were generated as follows: the DNA encoding LcpA and/or the extracellular domain of LcpA was amplified by PCR from *E. hirae* ATCC 9790 genomic DNA. The pET-22b (Novagen) was digested by *Nde*I and *Xho*I restrictions enzymes to remove the *pelB* leader region. When fused to a protein, this *pelB* leader sequence directs the protein to the bacterial periplasm. DNA fragments were cloned into this plasmid upstream the polyhistidine encoding sequence using the In-Fusion HD Cloning Kit (Clontech) as recommended by the supplier. The primers used in this study are listed in Table 2. Cloned sequences were verified by sequencing. Plasmids were named pET22b-*lcpA* and pET22b-*lcpA*_*30–293*_ respectively (Table 1).

**Table 2:** Primers used in this study

Primer	Sequence (5′ to 3′)	Application
32	ctttaagaaggagatatacatatgAAAACATTTCAAAAAGTGATTTTAGGTCTATTG	Cloning *lcpA* in pET-22b
33^a^	gtggtggtggtggtgTTGGTCTAAAAACTGATTGATTGCTAATTG	Cloning *lcpA* and *lcpA*_*30–293*_ in pET-22b
43	ctttaagaaggagatatacatatgTCTTCAACGAGTCGAGCAGACGATGTA	Cloning *lcpA*_*30–293*_ in pET-22b
CF9	CATGCCATGGCAAAAACATTTCAAAAAGTGATTTTAGGTCTATTG	Cloning *lcpA* in pCIPblaM
CF42^a^	CGAGCTCTTGGTCTAAAAACTGATTGATTG	Cloning *lcpA* in pCIPblaM

Reverse primers are indicated by ^a^. Underlined sequences are restriction sites engineered for cloning. Bases noted in small letters are homologous to bases of the linearized vector when using In-Fusion® HD Cloning Kit

For the determination of LcpA topology, we used the reporter gene *blaM* (encoding the mature form of the TEM β-lactamase) included in pCIPblaM plasmid [40]. In this plasmid the gene *blaM* was cloned under the control of the IPTG-inducible *lacUV5* promoter without its N-terminal signal peptide. The *lcpA* gene, without STOP codon, was amplified by PCR from *E. hirae* ATCC 9790 genomic DNA with primers listed in Table 2. The *lcpA* fragment thus obtained was cloned in the *Nco*I and *Sac*I restriction sites of the pCIPblaM vector to give pDML1677 bearing the *lcpA-blaM* in-frame fusion (Table 1).

### Protein expression and purification

The pET-22b-*lcpA* plasmid was introduced in *E. coli* Rosetta DE3 pLysS (Novagen) to overproduce the full-length LcpA protein (Table 1). Transformants were grown at 37 °C in LB medium supplemented with 100 μg ∙ ml^−1^ ampicillin with shaking. The overproduction of the protein was induced with 1 mM IPTG when the OD_600_ reached 0.3. Cells were then harvested by centrifugation, the supernatant was discarded and the cells were resuspended in the lysis buffer (20 mM Na_2_PO_4_, 100 mM NaCl, pH 7.0) and then disrupted with a French Press. Fractionation was performed by centrifugation and the full-length LcpA protein (membrane associated form) was obtained by solubilization of the membrane-fraction with CHAPS 0.3 % (AG Scientific). Solubilized LcpA was further purified by immobilized metal-ion affinity chromatography (IMAC) via the C-terminal His-tag fused to the protein. The elution was performed with a 0–500 mM imidazole gradient. The fractions containing the protein were dialyzed against 20 mM Na_2_PO_4_, 100 mM NaCl, 0,3 % CHAPS, pH 7.0, concentrated to 0,5 mg/ml and conserved in the same buffer at −20 °C, for no more that 1 month.

Similar conditions were used for the overproduction of the LcpA_30–293_ soluble form with the pET-22b-*lcpA*_*30–293*_ (Table 1). After the fractionation step, the soluble fraction was kept for purification of LcpA_30–293_. The purification protocol of LcpA_30–293_ was the same as above except that a second step of purification was needed to improve the purity of the protein. This additional step consisted of an ion-exchange chromatography with a MonoQ anion exchange column. This second step was performed in 20 mM Tris buffer pH 7.0 and protein was eluted with a NaCl gradient from 0 to 500 mM. The protein was dialyzed against 20 mM Tris, 100 mM NaCl, pH 7.0 concentrated to 5 mg/ml and conserved in the same buffer at −20 °C, for no more that 1 month.

LcpA and LcpA_30–293_ bands were isolated from SDS-PAGE. In situ digestion with trypsin followed by an analysis on an LC (nano Ultimate 3000 – Dionex) – ESI-ion trap (AmaZon-Bruker) in positive ion mode was performed. Mass spectroscopy of LcpA_30–293_ was performed on an ESI-Q-ToF (Waters, Micromass) in positive ion mode (Proteomic Platform, GIGA-Research Facility, University of Liège, Belgium).

### Purification of antibodies against LcpA_30–293_

Anti-serum against LcpA_30–293_ was raised in rabbit (CER, Marloie, Belgium). It was affinity-purified with LcpA_30–293_ immobilized on activated NHS-sepharose (GE Healthcare) column, as recommended by the manufacturer. The serum was loaded on the column, and after wash with PBS, the antibodies were eluted in 1 ml fractions of 50 mM glycine-HCl 150 mM NaCl pH 2.3, directly neutralized with 300 μl of 500 mM phosphate buffer pH 7.65.

The same protocol was followed for the purification of antibodies against PBP5, but using immobilized PBP5 protein (without signal peptide).

### Topology of LcpA in *E. coli*

*E. coli* TOP 10 F’/pDML1677 transformants were selected on LB kanamycin (50 μg ∙ ml^−1^). Then transformants were selected in patch onto LB agar plates containing IPTG (100 μM) and ampicillin (0–100 μg ∙ ml^−1^), following the method of Broome-Smith et al. [41].

### Western-blotting analysis of cellular fractions

Bacterial cells were disrupted in 20 mM Tris–HCl pH 7 with a French Press. Membrane and soluble fractions were separated by ultracentrifugation (1 h at 40,000 g). The proteins were separated by SDS-PAGE and electrotransferred to a PVDF membrane (Immobilon®-P Transfer Membrane, Millipore). The membrane was incubated in 3 % powder skimmed milk (weight/volume) with Tris-Buffer-Saline (TBS) at 4 °C overnight. Then incubation with primary antibodies (anti-LcpA in TBS-Tween) at dilution of 1/1000 and incubation with secondary antibodies (anti-Rabbit IgG - Alkaline Phosphatase antibody produced in goat, Sigma-Aldrich) were realized. The detection is done with NBT (nitro blue tetrazolium) + BCIP (5-bromo-4-chloro-3-indolyl-phosphate)/HCO_3_ buffer pH 9.5.

### Immunofluorescence microscopy

The protocol from Morlot et al. [42] was slightly modified to observe enterococci. Briefly, cells grown in BH medium were harvested at OD_600_ = 0.3, and fixed in PBS buffer containing 4 % paraformaldehyde (volume/volume) for 30 min at room temperature and 1 h on ice. The fixed cells were washed 3 times with PBS and resuspended in 50 mM glucose, 20 mM Tris–HCl pH 7.5, 10 mM EDTA and 1 mg ∙ ml^−1^ lysozyme. Cells were directly poured onto poly L-lysine coated Poly Prep™ slides (Sigma). Then the slides were dipped in methanol at −20 °C for 5 min and air-dried for 3 min. The slides were rehydrated and blocked with 2 % (weight/volume) bovine serum albumin in PBS (PBS-BSA) for 1 h at room temperature. Cells were incubated for 1 h with a 1:100 dilution of rabbit polyclonal anti-LcpA or anti-PBP5 antibodies in PBS-BSA and washed 10 times with PBS. Samples were then incubated with a 1:100 dilution of Cy3-conjugated goat anti-rabbit immunoglobulins G (Jackson Immunoresearch Laboratories) in PBS-BSA for 1 h at room temperature in the dark. A fluorescent derivative of the antibiotic vancomycin (Vancomycin, BODIPY® FL conjugate, Invitrogen Molecular Probes) was used as a probe for nascent peptidoglycan and was added with the secondary antibody. For optimal staining, a 1:1 mix of fluorescent vancomycin and unlabeled vancomycin at a final concentration of 1 μg ∙ ml^−1^ was realized as described before [43, 44]. For DNA staining, 2 μg ∙ ml^−1^ of 4’,6-Diamidino-2-phenylindole (DAPI, TEBU-Bio) were added. After 10 washes with PBS, the slides were mounted with Mowiol and examined with an Olympus BX61 microscope endowed of an UPFLN 100× O-2PH/1.3 objective and a QImaging Retiga-SRV 1394 cooled charge-coupled device camera. Image acquisition was performed using the Volocity 6 software.

### LcpA-PG interaction

PG from *E. hirae* ATCC 9790 with and without SCWP was prepared as described previously [45]. Briefly, a 2 L culture in Brain Heart Infusion broth (Difco) was incubated at 37 °C until OD_600_ of 0.5. Cells were harvested by centrifugation for 10 min at 4 °C at 7,500 g and resuspended in 40 ml of ice-cold water. The cell suspension was poured dropwise into 40 ml of boiling 8 % sodium dodecyl sulfate (SDS) and boiled for 45 min. Insoluble peptidoglycan was pelleted by centrifugation at 20 °C for 20 min at 100,000 g. The pellet was washed with water until it was free of SDS (Hayashi, 1975). All centrifugation steps were performed at 100,000 g. The pellet was resuspended in 40 ml of 7 mM NaCl, 20 mM phosphate buffer, pH 6.9 and 200 μg/ml of α-amylase (final concentration) were added. Samples were incubated for 3 h at 20 °C, with gentle shaking and then centrifuged for 60 min at room temperature. The pellet was resuspended in 40 ml of 100 mM Tris, pH 8.0 and 200 μg/ml of trypsine (final concentration) were added. Samples were incubated for 18 h at 25 °C with gentle shaking and then centrifuged for 60 min at room temperature. The pellet was resuspended in 40 ml of 100 mM Tris, pH 7.5 and 500 μg/ml of pronase were added. Samples were incubated for 3 h at 40 °C. and then centrifuged for 60 min at room temperature. The pellet was washed 3 times with cold water. At this step, samples were split and half of the pellet was treated with 49 % hydrofluoridic acid during 48 h at 4 °C to remove SCWPs. Hydrofluoridic acid was removed by centrifugation and both cell wall preparations, the acid-treated and the non-treated one, were washed twice with 20 ml of 8 M LiCl, and then twice with 20 ml of 100 mM EDTA pH 8.0. Cell walls were finally washed 3 times with water before being resuspended in 2 ml of water, freeze-dried and conserved at 4 °C for further utilisation. The interaction between LcpA and purified *E. hirae* PG was analyzed as described by Kerff et al. [46] with slight modifications. Briefly, 20 μg of purified LcpA were incubated on ice for 10 min with 6 mg of purified insoluble peptidoglycan from *E. hirae* ATCC 9790, with or without SCWP, in 500 μl of water. Then the samples were centrifuged for 10 min at 13,000 g, the supernatant was separated, and the pellet was washed once with 1 ml of water. These two first supernatants were pooled (sample 1). The pellet was further incubated for 10 min on ice with 500 μl of 5 M NaCl. The supernatant was recovered by centrifugation, and a second wash with 1 ml of the same solution was performed. Both supernatants were pooled (sample 2). The pellet was incubated with 500 μl of 2 % SDS at 100 °C for 5 min. The sample was centrifuged, the supernatant recovered, and the treatment was repeated with 1 ml of 2 % SDS. Both supernatants were pooled (sample 3). The pellet was then resuspended with 50 mM phosphate buffer pH 7 and dialyzed overnight at 4 °C against the same buffer. The sample was adjusted to a 1.5 ml volume, supplemented with 4 μg of lysozyme, and digested overnight at 37 °C (sample 4). For each sample (1 to 4) an equivalent volume of 300 μl was concentrated, heated in denaturing buffer (10 % glycerol, 2.5 % 2-mercapto-ethanol, 1 % SDS, 31.25 mM Tris–HCl, 0.003 % bromophenol blue pH 6.8). The samples were resolved by SDS-PAGE and estimated visually with Coomassie blue staining.

## Results and discussion

### *Enterococcus hirae* LCP genes

**Fig. 1:**
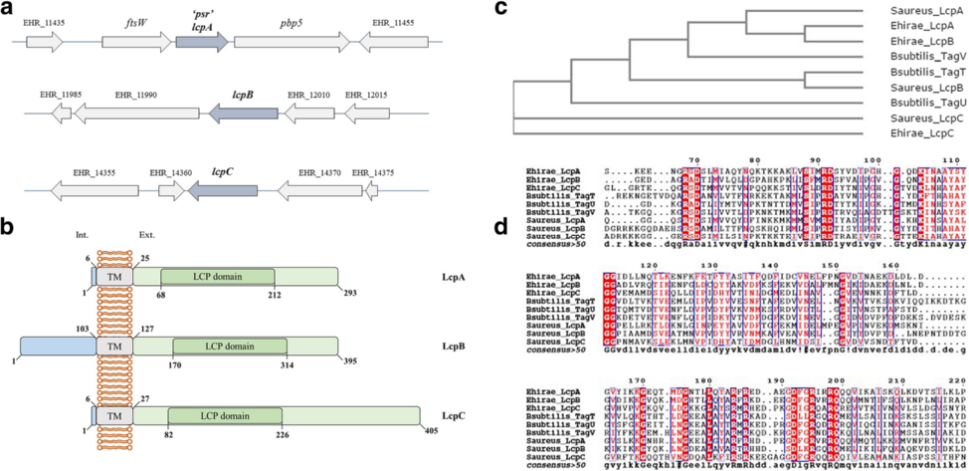
**a** Genetic organization of the 3 *lcp* gene loci in the genome of *E. hirae* ATCC 9790. Dark shaded arrows represent the *lcp* genes. The *psr* gene is here renamed *lcpA* and takes place between *ftsW* and *pbp5* genes. Other genes are annotated as: nitrilotriacetate monooxygenase component B (EHR_11435); major facilitator superfamily permease (EHR_11455); hypothetical protein (EHR_11985); topoisomerase IA (EHR_11990); shikimate 5-dehydrogenase (EHR_12010); ABC transporter ATP-binding protein (EHR_12015); chitinase B (EHR_14355); tspO protein (EHR_14360); UDP-N-acetylmuramyl pentapeptide synthase (EHR_14370); hypothetical protein (EHR_14375). Annotation follows NCBI available sequence data for *E. hirae* ATCC 9790. **b** Predicted domains of *E. hirae* LCP proteins. The membrane topology was predicted for each LCP proteins with the TMHMM Server v.2.0. The LCP domains were predicted using the pfam03816 consensus sequence. **c** Phylogenetic tree, based on the multiple alignment of LCP proteins from *E. hirae*, *B. subtilis* and *S. aureus*, showing the relatedness of LCP proteins from these firmicutes bacteria. The tree was calculated with Clustal W. **d** Alignment of the LytR-CpsA-Psr domains of LCP proteins from *E. hirae*, *B. subtilis* and *S. aureus*. Full length sequences were aligned using Clustal W and formatted with ESPript, only the LCP domain as defined in the PFAM database (pfam03816) is shown. The red color is depending on the identity of residues. The white color on a red background denotes strictly conserved residues. A consensus sequence based on the aligned sequences is shown in the lower row. From the consensus sequence, the following pattern has beed deduced R-X-D-X(20)-R-D-X(91,103)-R-X-R-X(4,7)-D-X(2)-R-X(2)-R-Q (Prosite syntax). These residues are highly conserved in LCP proteins and, in known LCP 3D-structures, their side-chains are spatially close, most of them being involved in the binding of the pyro-phosphate lipid [28] (for more details see Additional file 1: S2)

Many Gram-positive organisms contain several members of the LCP family of proteins. BLAST searches, using amino acids sequences of LcpA (Psr) from *E. hirae* and the LCP consensus sequence (pfam03816) as queries, allowed to identify 3 homologues in the genome of *E. hirae* ATCC 9790 (10). These three members of the LCP family are: EHR_11445/AFM71168.1, EHR_11995/AFM71268.1, EHR_14365/AFM71716.1. It is worth mentioning that when EHR_11995/AFM71268.1 was used as query sequence for BLAST search through NCBI protein data-base, EHR_11995/AFM71268.1 sequence appeared N-terminal truncated in the *E. hirae* ATCC 9790 genome (10) as compared with other *E. hirae* sequences available in the NCBI data-base. Thus, the corresponding region of the *E. hirae* ATCC 9790 genome was sequenced. The corrected sequences used for further analysis are listed in (Additional file 1: S1). The three members of the LCP family are annotated « Cell envelope-associated transcriptional attenuator », « Transcriptional regulator, Psr protein », and « Transcriptional regulator » respectively [12]. Unfortunately, the regulatory role previously attributed to these proteins remains upon their names, leading to confusion about their actual role. We propose to rename EHR_11445, EHR_11995 and EHR_14365 as LcpA (formerly Psr protein), LcpB and LcpC, respectively (Fig. 1a).

A common topology arises for the three LCP proteins from *E. hirae* when amino acid sequences are analyzed *in silico* with the TMHMM program (Fig. 1b). TMHMM predicts the presence of a N-terminal cytoplasmic tail of different length depending on the protein, (6 amino acids for LcpA, 103 amino acids for LcpB and 6 amino acids for LcpC), followed by a transmembrane α-helix of ~ 20 amino acids (18, 23 and 20 amino acids respectively), and an LCP C-terminal domain located in the extracellular space. No other functional conserved domain could be identified (Table 3).

**Table 3:** Amino acid sequence analyses of LytR-CpsA-Psr proteins and prediction of transmembrane regions

	*E. hirae*_LcpA	*E. hirae*_LcpB	*E. hirae*_LcpC	*B. subtilis*_TagT	*B. subtilis*_TagU	*B. subtilis*_TagV	*S. aureus*_LcpA	*S. aureus*_LcpB	*S. aureus*_LcpC
Number AA	293	395	405	322	306	391	327	405	307
Molecular weight (Da)	32720.2	44259.3	45409.3	35921.2	34586.4	43211.3	36970.7	45685.2	33804.4
Isoelectric point	4.99	9.64	4.91	9.16	9.18	8.78	9.66	6.02	7.86
TMHMM	7–24	104–126	7–26	20–41	13–35	20–39	32–54	7–28	7–28

The amino acid sequences of the full length proteins were analyzed using ProtParam. Transmembrane segments were predicted using the web-based server TMHMM. Positions of the first and the last residues of the predicted transmembrane domains are indicated

A multiple-sequence alignment and its associated dendrogram were realized using Clustal W to compare different LCP proteins from the Gram-positive species *E. hirae*, *B. subtilis* and *S. aureus* (Fig. 1c and d; and Table 4). These species contain three LCP homologues each. The consensus sequence of the LCP domains showed highly conserved charged residues, probably related to the active site of the protein, as already observed in previous phylogenetic study [24]. Figure 1c and Table 4 indicate that LcpA and LcpB from *E. hirae* are highly related (46 % identity), LcpA from *E.hirae* is close to LcpA from *S. aureus* (42 % identity)*,* an enzyme that attaches teichoic acids to the peptidoglycan*.* LcpC appears less close to the *E. hirae* homologues than to those of *B.subtilis* and *S. aureus*. Evidences suggest that *tagV* from *B.subtilis* would be required for teichoic acid accumulation in the wall whereas *lcpC* from *S. aureus* encodes the key enzyme for immobilization of capsular polysaccharide to the staphylococcal cell wall. In *B. subtilis*, the genetic environment of *lcp* genes (*tag* genes) is related to synthesis and/or translocation to the outside of the cell of SCWP (teichoic acids or capsular polysaccharides). In *E. hirae*, instead, the regions surrounding *lcp genes* do not include genes related to SCWP. Paralogues of these genes are located in other regions of the genome, far from the *lcp* genes.

**Table 4:** Cross-study comparison of the nine LCP proteins from *E. hirae*, *B. subtilis* and *S. aureus*

	*E.hirae*_LcpA	*E.hirae*_LcpB	*E.hirae*_LcpC	*B.subtilis*_TagT	*B.subtilis*_TagV	*B.subtilis*_TagU	*S.aureus*_LcpA	*S.aureus*_LcpB	*S.aureus*_LcpC
	293 AA	395 AA	405 AA	322 AA	306 AA	391 AA	327 AA	405 AA	307 AA
*E.hirae*_LcpA		292/293	246/293	183/293	231/293	200/293	243/293	271/293	182/293
		E = 7.10^−85^	E = 1.10^−33^	E = 3.10^−33^	E = 4.10^−31^	E = 1.10^−28^	E = 1.10^−68^	E = 3.10^−22^	E = 8.10^−30^
*E.hirae*_LcpB	135/292		211/395	160/322	160/306	182/391	257/327	156/395	146/307
	46,23%		E = 2.10^−23^	E = 1.10^−23^	E = 6.10^−28^	E = 6.10^−23^	E = 3.10^−67^	E = 2.10^−19^	E = 3.10^−21^
*E.hirae*_LcpC	78/246	63/211		295/322	279/306	289/391	184/327	270/405	289/307
	31,71%	29,85%		E = 5.10^−46^	E = 1.10^−79^	E = 2.10^−42^	E = 1.10^−26^	E = 4.10^−40^	E = 7.10^−61^
*B.subtilis*_TagT	74/183	56/160	101/295		281/306	286/322	156/322	262/322	301/307
	40,43%	35%	34,24%		E = 2.10^−50^	E = 1.10^−52^	E = 8.10^−25^	E = 1.10^−57^	E = 2.10^−33^
*B.subtilis*_TagU	78/231	58/160	118/279	106/281		280/306	209/306	276/306	289/306
	33,77%	36,25%	42,29%	37,72%		E = 4.10^−63^	E = 1.10^−28^	E = 3.10^−44^	E = 2.10^−74^
*B.subtilis*_TagV	72/200	64/182	92/289	109/286	116/280		184/327	289/391	305/307
	36%	35,16%	31,83%	38,11%	41,43%		E = 2.10^−30^	E = 1.10^−47^	E = 3.10^−43^
*S.aureus*_LcpA	103/243	112/257	64/184	59/156	68/209	72/184		189/327	188/307
	42,39%	43,58%	34,78%	37,82%	32,54%	39,13%		E = 9.10^−21^	E = 8.10^−25^
*S.aureus*_LcpB	79/271	55/156	88/270	106/262	95/276	101/289	60/189		263/307
	29,15%	35,26%	32,59%	40,46%	34,42%	34,95%	31,75%		E = 4.10^−38^
*S.aureus*_LcpC	69/182	47/146	108/289	89/301	120/289	95/305	65/188	86/263	
	37,91%	32,19%	37,37%	29,57%	41,52%	31,15%	34,57%	32,70%	

The comparison was realized with « blast2seq » using default settings. The values below the main diagonal represent, respectively, the ratio between the conserved residues and the length of the smallest sequence aligned without gap and the percentage of identity between both sequences. The values above the main diagonal represent, respectively, the ratio between the number of residues of the shortest sequence aligned without gap and the number of residues of the shortest sequence before alignment, and the expected « E » value

The *lcpA* gene (Fig. 1a) is located between the *ftsW* gene, coding for a SEDS (Shape, Elongation, Division and Sporulation) protein involved in Lipid II export, and the *pbp5* gene, coding for a low-affinity PBP involved in peptidoglycan synthesis. These three genes, *ftsW-lcpA-pbp5,* are organized into an operon, since a polycistronic mRNA including the three genes could be isolated from *E. hirae* and *E. faecium* [20]*.* Unlike the observations made in other species for LCP proteins, *lcpA* is not flanked by any cluster of known SCWP synthetic genes.

### Cloning and expression of LcpA

**Fig. 2:**
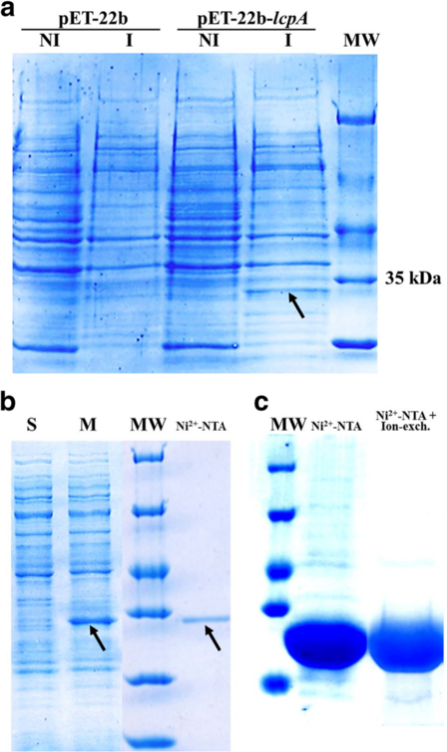
**a** SDS-PAGE analysis of LcpA overproduction in *E. coli* Rosetta DE3 pLysS cells. Total fractions of induced (I) and non induced (NI) cultures transformed with pET-22b or pET-22b-*lcpA* underwent electrophoresis. The arrow shows the overproduction of LcpA. MW – Molecular Weight Marker. **b** SDS-PAGE analysis of the soluble (S) and membrane (M) fractions. Purification of recombinant LcpA by Ni^2+^-NTA chromatography (Ni^2+^-NTA). The arrows show the LcpA protein. MW – Molecular Weight Marker. **c** Purification of recombinant LcpA_30–293_. SDS-PAGE analysis after first step of purification by Ni^2+^-NTA chromatography (Ni^2+^-NTA) and after second step of purification by ion-exchange chromatography (Ni^2+^-NTA + Ion exch.). MW – Molecular Weight Marker

The pET-22b-*lcpA* plasmid was constructed by removing the *pelB* leader region to express the protein within the cytoplasm, and cloning the *lcpA* gene from *E. hirae* ATCC 9790 encoding the full length protein (including the membrane-associated domain) [23]. The resulting construction was verified by sequencing and then transformed into different expression strains. Many different conditions (including expression strains, incubation temperatures, induction times) were tested before membrane-associated protein could be produced in a detectable amount. The best conditions to produce LcpA were found when 2.5 l culture of *E. coli* Rosetta DE3 pLysS at 37 °C, were induced with 1 mM of IPTG when the OD_600_ reached 0.3. A band migrating at a position compatible with the molecular weight of the LcpA protein (32.7 kDa) could be detected by Coomassie blue staining after the separation of cellular proteins by SDS-PAGE (Fig. 2a). Cellular fractionation highlighted that LcpA was mostly detected in the membrane fraction of the cellular lysate (data not shown). Addition of 0.3 % CHAPS to the cellular lysate solubilized the membrane and allowed the detection of LcpA in the solubilized fraction (Fig. 2b). The protein from a solubilized membrane-fraction could be purified to homogeneity by IMAC through the C-terminal His-tag fused to the LcpA protein. The overall production and purification yielded 300 μg of protein from 2.5 l of culture (Fig. 2b). The identity of the protein was confirmed by peptide mass fingerprinting. Thus, a reproducible protocol of production and purification of the entire membrane-associated LcpA protein of *E. hirae* was established. In addition, we observed that in *E. coli* Rosetta DE3 pLysS producing LcpA, the protein was localized in the membrane fraction. This result is in agreement with the *in silico* transmembrane helix topology prediction.

As the production yield for the membrane-associated LcpA was rather low, a LcpA construct deleted from its transmembrane helix was produced to raise and purify antibodies needed for further studies. The sequence corresponding to the extracellular domain of LcpA (containing amino acids 30–293) was cloned in the pET-22b plasmid. Overproduction of LcpA_30–293_ was performed in the conditions used for producing the full-length LcpA. LcpA_30–293_ was purified to homogeneity by successive IMAC and an ion-exchange chromatography steps (Fig. 2c). Production and purification yields for LcpA_30–293_ were higher than for LcpA, and we obtained ~30 mg of LcpA_30–293_ starting from 2.5 l of bacterial culture. The identity of the protein was confirmed by mass spectrometry (in the experimental conditions, LcpA_30–293_ was not associated with any other molecule). Polyclonal antibodies against LcpA_30–293_ were generated in rabbits. The sera were affinity-purified against the LcpA_30–293_ protein as described in the materials and methods section in order to obtain specific antibodies against LcpA.

### Characterization of LcpA topology in *E. coli*

The reporter gene *blaM* encoding the mature form of the TEM β-lactamase has been extensively used to characterize membrane protein topology [47]. This β-lactamase provides an ampicillin-resistant phenotype at single-cell level when exported to the cell periplasm [40]. In-frame fusion of LcpA to TEM was made by generating the pDML1677 (Table 1). In this plasmid, the *blaM* gene lacks its own signal peptide, thus, a colony with a resistant phenotype, at single cell level could only be observed if BlaM is expressed in the periplasm due to the LcpA signal peptide. The plasmid was introduced in *E. coli* TOP 10F’ and transformants were selected on kanamycin. Transformants were then transferred “in patch” onto LB agar plates containing IPTG, kanamycin and ampicillin to select plasmid exhibiting LcpA-blaM in frame fusion. All the clones that grew as single-cell colonies at high ampicillin concentration (100 μg ∙ ml^−1^) showed that the *lcpA-blaM* fusion exerted a protective effect against the ß-lactam, consistent with the presence of LcpA signal peptide directing BlaM β-lactamase to the periplasm. In contrast, plasmid free or pCIPblaM transformed *E. coli* controls failed to grow in the presence of ampicillin above 5 μg ∙ ml^−1^ (Table 5). Results of gene fusion experiments in *E. coli* cells further confirm *in silico* prediction indicating that *E. hirae* LcpA signal peptide is not cleaved by signal peptidase and that it allows the anchoring of the protein to the cytoplasmic membrane. Therefore, *E. hirae* LcpA is another example of an extracellular membrane-bound member of the LytR/CpsA/Psr family.

**Table 5:** Study of LcpA topology: LcpA-BlaM hybrid characterization for antibiotic single cell resistance

	Single cell test	
Strains	*E. coli* Top10/pDML1677	*E. coli* Top10 F’/pCIPblaM
Characteristics	*lcpA-blaM* hybrid	Δ *blaM*
Km^a^	Resistant	Resistant
Km^a^ + Amp^b^ + IPTG^c^	Resistant	Sensitive
MIC-Value (μg.ml^−1^)	>100	2
β-lactamase moiety location	Outside the cell	-

^a^ Kanamycin : 50 μg.ml^−1^, ^b^ Ampicillin: 10 μg.ml^−1^, ^c^ IPTG: 100 μM

### LcpA localization in *Enterococcus hirae*

**Fig. 3:**
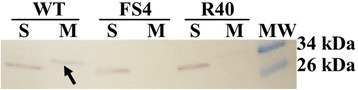
Western blot analysis with antibody against LcpA. Soluble (S) and membrane (M) fractions of different *E. hirae* strains were investigated: *E. hirae* ATCC 9790 wild-type (WT) and mutants for *lcpA* (FS4 and R40) as negative controls. The arrow shows the LcpA protein. MW – Molecular Weight Marker

A preliminary localization study of LcpA was performed on soluble and membrane fractions of *E. hirae* lysates incubated with purified polyclonal antibodies against LcpA. Western blot analysis showed that LcpA is mainly localized in the membrane fraction of *E. hirae* ATCC 9790 (Fig. 3). On the contrary, no LcpA signal was neither detected in the *E. hirae* R40 mutant, in which the *lcpA* open reading frame is disrupted [7], nor in the FS4 mutant, in which, the *lcpA* gene has been replaced by a kanamycin resistance cassette [22]. It should be noted that a slight gel shifting effect is observed for LcpA band contained in membrane fraction. The identity of this band was verified by peptide-mass fingerprinting. Besides*,* another signal can be observed in the three soluble fractions at about 34 kDa. Although the polyclonal antibodies have been purified from rabbit serum with LcpA as a bait protein, we cannot exclude cross-reaction with other epitopes when polyclonal anti-LcpA antibodies are used.

### Binding of LcpA to peptidoglycan

**Fig. 4:**
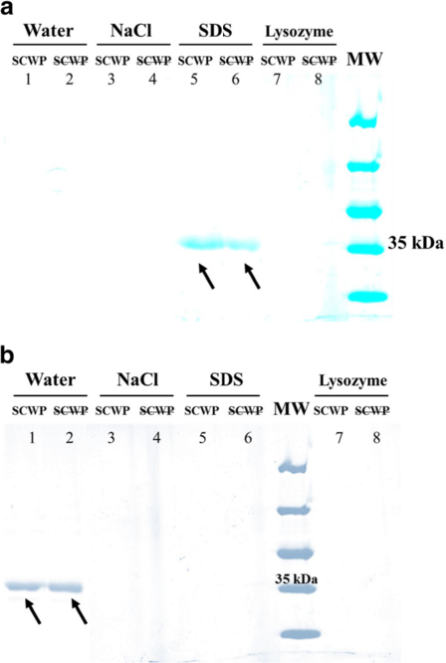
**a** Binding of LcpA to *E. hirae* cell wall with (SCWP) or without () secondary cell wall polymers. SDS-PAGE analysis of supernatant after water wash of LcpA-cell wall complex (Water), supernatant after 5 M NaCl wash of LcpA-cell wall complex (NaCl), supernatant after 2 % SDS wash of LcpA-cell wall complex (SDS), and supernatant after lysozyme digestion of LcpA-cell wall complex (lysozyme). The arrows show the LcpA protein. MW – Molecular Weight Marker. **b** The experience was done under identical conditions but using *B. subtilis* cell wall (control)

The interaction of full-length LcpA with *E. hirae* cell wall was investigated by pull-down experiments. Purified cell wall from *E. hirae*, containing peptidoglycan with or without SCWP was incubated with LcpA. Centrifugation allowed to separate the insoluble material, containing the purified cell wall and the proteins bound to the peptidoglycan or to the SCWP, and the soluble fraction, containing the unbound proteins. The soluble fraction was analyzed by SDS-PAGE (Fig. 4a). The absence of LcpA in lane 1 indicates that the protein has been pelleted with the insoluble fraction of the reaction mix and is thus able to bind the cell wall. The absence of LcpA in lane 2 further indicates that LcpA is still present in the insoluble fraction of the reaction mix in the absence of SCWP. LcpA thus likely binds to the peptidoglycan moiety of the cell wall, however, because the chemical structure of enterococcal SCWP is poorly known, the ineffectiveness of 49 % HF to completely eliminate all SCWP cannot be excluded. 5 M NaCl did not allow to release LcpA from the cell wall (lanes 3–4). Heating the complex with 2 % SDS released LcpA from the cell wall and a similar amount of protein was released in the presence and after removal of SCWP, further demonstrating that LcpA is able to bind peptidoglycan (lanes 5–6). The treatment with lysozyme, which intended to solubilize the peptidoglycan and thus release in the soluble fraction any peptidoglycan-protein complex that would have resisted the SDS treatment, did not release more material in the soluble fraction (lanes 7–8). Similar experiments realized with *Bacillus subtilis* peptidoglycan showed no interaction (Fig. 4b). These results indicate that LcpA binds to the peptidoglycan moiety of the cell wall through non-ionic interactions and that this interaction could be specific to enterococcal Lys-type peptidoglycan.

### Immunolocalization of LcpA in *Enterococcus hirae*

**Fig. 5:**
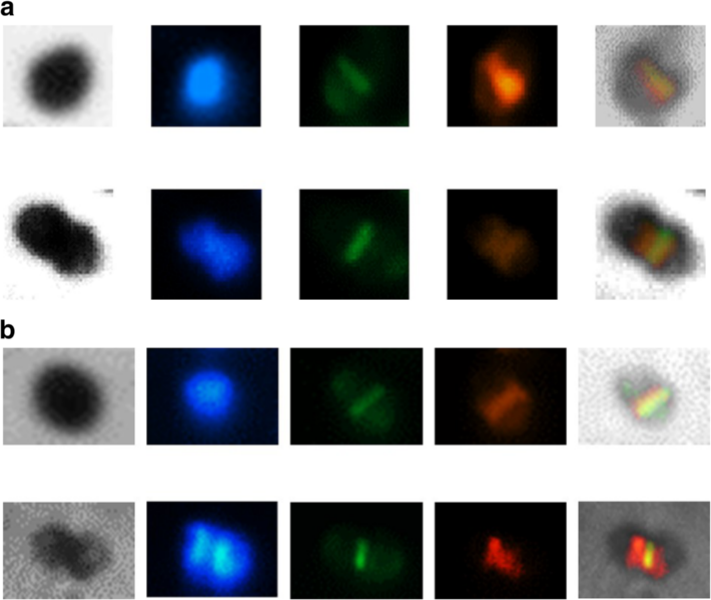
LcpA (**a**) and PBP5 (**b**) immunolocalization in *E. hirae* cells. Cells were stained for DNA with DAPI (blue), peptidoglycan synthesis regions with fluorescent vancomycin (green), LcpA with anti-LcpA (**a**) or PBP5 with anti-PBP5 (**b**) primary antibodies and Cy3-conjugated secondary antibodies (red). The upper panels show cells at an early division stage, as indicated by the presence of a single nucleoid and no apparent sign of midcell constriction. The lower panels show cells at a later stage of division, as indicated by the presence of a double nucleoid and a clear sign of midcell constriction

The cellular localization of LcpA, was assessed by immunofluorescence microscopy. *E. hirae* ATCC 9790 cells in exponential phase (OD_600_ ~ 0.3) were fixed, permeabilized and incubated with purified rabbit polyclonal antibodies directed against LcpA. A secondary anti-rabbit goat antibody coupled to Cy3 was used to visualize the localization of LcpA, regions of peptidoglycan synthesis were labeled using fluorescent vancomycin and DNA was labeled using DAPI (Fig. 5a). In pre-divisional cells displaying a single nucleoid (Fig. 5a, upper panels), LcpA co-localizes with nascent peptidoglycan at midcell. In cells undergoing constriction and displaying partially segregated nucleoids, LcpA appears as two bands flanking the central band of nascent peptidoglycan (Fig. 5a, lower panels). In these cells, LcpA might thus localize to the division sites of the future daughter cells. In the R40 mutant (*lcpA* ^*−*^), which does not express LcpA, no fluorescence signal was observed (Additional file 1: S3), indicating that the fluorescent signal observed in the wild type cells is specific to LcpA localization. The localization of LcpA at the parental division site and later, at the division sites of the future daughter cells suggests that, like several other proteins of the LCP family, LcpA could play a role in cell wall metabolism, attaching polymers to the peptidoglycan. The genetic localization of the *lcpA* gene, which is placed between the *ftsW* and *pbp5* genes in the genome of *E. hirae*, also support the idea that LcpA might be involved in the peptidoglycan metabolism. We investigated the localization of PBP5 by immunofluorescence microscopy, using purified rabbit polyclonal antibodies directed against PBP5. Interestingly, PBP5 displayed a localization pattern similar to that of LcpA. Indeed, PBP5 localized at the parental division site in pre-divisional cells (Fig. 5b, upper panels) and at the division sites of the future daughter cells in cells undergoing constriction (Fig. 5b, lower panels). These immunofluorescence studies suggest that the two proteins localize in similar regions in *E. hirae* cells, and might thus have related functions in the cell wall. In our hands, however, any attemp to show interaction between these two proteins failed (unpublished results).

## Conclusion

The results obtained in this work show that LcpA belongs to the LCP family, that it is a membrane protein that binds peptidoglycan, and localizes, together with PBP5, at sites flanking nascent peptidoglycan. However, its role remains unknown. The observations published several years ago about the changes in the CW polymers observed in the R40 mutant that possesses a truncated *lcpA* gene may be relevant in the light of the recent findings. This mutant showed a decreased content of CW rhamnose, not related to the overproduction of PBP5 nor to other changes in the peptidoglycan structure [19]. Thus, a tempting hypothesis could be that LcpA catalyzes the attachment of “rhamnose-containing polysaccharides” from the flipped lipid-carrier onto the peptidoglycan of *E. hirae,* as suggested in a recent publication describing rhamnose-containing polysaccharides [39]*.* It is noteworthy, however, that LCP-coding genes often localize in close proximity to the gene cluster encoding the secondary polymers for which they exert their catalytic effects [30] as is the case for *B. subtilis tagTUV* [26], *S. pneumoniae cps2A*, *lytR* and *psr* [28]. Like *lcpABC* from *S. aureus* [30], the *lcpA* from *E. hirae* (and also from *E. faecium*) does not seem to be part of any biosynthetic cluster. Extensive work should be done to clearly identify all the genes involved in the enterococcal rhamnose-containing polysaccharides synthesis in order to determine if LcpA, as other members of the LCP family, acts as a phosphotransferase catalyzing the attachment of rhamnose-containing polysaccharides to the peptidoglycan of *E. hirae*.

## Additional file

Additional file 1: Supplemental data. (DOCX 226 kb)

## Abbreviations

CW: Cell wall
LCP: LytR-CpsA-Psr
PBP: Penicillin-binding protein
PG: Peptidoglycan
SCWP: Secondary cell wall polymers
WTA: Wall teichoic acids
