# Supplementary material for: Enterococcus hirae LcpA (Psr), a new peptidoglycan-binding protein localized at the division site
Source: BMC Microbiol. 2016 Oct 12;16:239. doi: 10.1186/s12866-016-0844-y (PMC5059904; doi:10.1186/s12866-016-0844-y)
Supplement: Additional file 1: — Supplemental data. (DOCX 226 kb) [file 12866_2016_844_MOESM1_ESM.docx]

# Supplemental data

S1

The nucleic acid and amino acid sequences of the 3 LCP from *Enterococcus hirae* ATCC 9790. The *lcp* genes from *E. hirae* were sequenced in this study and present several differe nces compared to those found within the *E. hirae* genome CP003504.1 in the NCBI database.

For the amino acid sequences of the LCP proteins from *Bacillus subtilis* and *Staphylococcus aureus* the accession number is indicated.

> *Enterococcus hirae lcpA* gene sequence ATGAAAACATTTCAAAAAGTGATTTTAGGTCTATTGACGGCAGTCGTCATTGTCATCGGC TATTTTTCTTTTGAGTTGGATCATGGTTATTCTTCAACGAGTCGAGCAGACGATGTACCA AAAGTAGAAGCCAAAAGTGTTCCTAAGATTATCAATGTGGCATTGATTGGCTCTGATGCC CGGTCGAAAGAAGAAAATGGACGTTCAGATTCTTTGATGATTGCTCAGTATAATCAAAAA ACTAAAAAAGCAAAATTGGTTTCGATTATGCGTGATTCTTATGTAGATATACCTGGGCAT GGTCAAGATAAGATCAACGCAGCTTATTCATATGGAGGGATTGATCTTTTGAACCAAACA TTGAAAGAAAATTTCAAATTTGAAACACCTTATTATGCAAGTATCACCTTCCAAGATTTC ATTGATTGTGTCAATGAGCTATTCCCTAACGGTGTAGATATCAATGCTGAGAAAGATTTA GACTTGGATGGTGTATACATCAAAAAAGGTGAACAAACGATGGATGGGAATACCTTATTG CAATATGCGCGTTTTAGAGAAGATGCTGAAGGAGATTTTGGACGGATCAGAAGACAACAA CAAGTGATCAAAGCAATTTCTAAGCAATTAAAAGATGTGACCTCCATCCTTAAATTACCA AAAGCAATCGGTCAACTCTTGGGAAGTATTAAAACCAATTTACCTGAAAGTGTATTAATT GATTGTGGCATTGATTTCTTAAAAGATGACAAACCAATTGATACGTTATCTGTACCAGTT GAAGGTAGTTGGAATTTTAATGATTACACACCTTCTGGTAGTGTACTAGAACTTGATTTA GAAAAAAATCAATTAGCAATCAATCAGTTTTTAGACCAATAG

> *Enterococcus hirae* LcpA amino acid sequence MKTFQKVILGLLTAVVIVIGYFSFELDHGYSSTSRADDVPKVEAKSVPKIINVALIGSDA RSKEENGRSDSLMIAQYNQKTKKAKLVSIMRDSYVDIPGHGQDKINAAYSYGGIDLLNQT LKENFKFETPYYASITFQDFIDCVNELFPNGVDINAEKDLDLDGVYIKKGEQTMDGNTLL QYARFREDAEGDFGRIRRQQQVIKAISKQLKDVTSILKLPKAIGQLLGSIKTNLPESVLI DCGIDFLKDDKPIDTLSVPVEGSWNFNDYTPSGSVLELDLEKNQLAINQFLDQ

> *Enterococcus hirae lcpB* gene sequence ATGAGTCGGATGGATCGTTATAAGAACATCCATAAAAAAGCAAAACCATTAAAAAAAGAA TCAACCGGTTTTTTTAGACGTGAACGTCAAAAGGACAAAGCGTTGAATGATCCAAGAGAT GAAGCTACTCGGATCTATCAACCTGATCAGGATTCTTCTTATTCTTCCAGAGAAGGACAA GCTAATGACTATCAAACCCCTTATTCCTATGCAAAAGCAGGAAACAAAAAAGGATTTAAT CAAACGAAGAAAAATTTTTTTAAGAGGAAGAAAGAGCCAAAAGAAGTTCGTCCAAAAAAG AAACGTTCGTGGGGGAAAATCATTTTAGGCATTATTCTATTTTTATTCTTATTTTCAGTC ATTTCCTTTTTTGTCGGGAAATCCATGGCAGAGCATGATACCAGTTTGCCGAGTGTTACA ACGGAATCATTTGATGGTGTACAGTCTTCGAGTGGCGCACATAATATTTTGATATTAGGT AGTGACACTAGAGGTGAGGATGCTGGTCGTGCAGATACGATCATGGTGTTACAGTTAGAT GGACCTGCTCATAAACCAAAATTGATCTCATTCATGCGTGATAGTTTTGTAGCAATTCCA GGAGTTGGTCAAAACAAGATCAATGCAGCTTATGCATATGGCGGTGCTGATCTGGTCCGT CAAACCATCAAAGAAAATTTTGGACTTGATTGCCAGTATTACGCAAAAGTCGATTTTAAA TCGTTTGAAAAAGTGGTCGATGCATTGTTTATGAATGGTATCAAGATCGATGCAGAGAAA GATTTGAATCTGGATGGTGTCGATATCAAAAAAGGTGTCCAAAAAATGGATGGTCATACA CTTTTACAGTATGCACGTTTTAGAAAAGACGAAGAAGGCGATTTTGGTCGAGTAAGACGG CAACAACAAGTCATGAATACGATTTTTAGTCAGCTAAAAAATCCCCTAAACTTGATTCGT GCCCCTTACGCTGCCGGAAAAGCCATTGGTTATACCTCAACGGATGTTTCATCATTCTTT ATTATCAAAAATCTCTTGTCGATTGCTCGAGGTGTCGGTGGTGTGGATCGCTTAAGTGTG

CCTGTTGAAGGTTCATGGAACTTCGGAAACAGTAGTTATGCCGGAAGTATTTTGGTGATC GACAATGACGCCAATCGTGCAGCAATCAGTGATTTTTTAAGTAAATAA

> *Enterococcus hirae* LcpB amino acid sequence MSRMDRYKNIHKKAKPLKKESTGFFRRERQKDKALNDPRDEATRIYQPDQDSSYSSREGQ ANDYQTPYSYAKAGNKKGFNQTKKNFFKRKKEPKEVRPKKKRSWGKIILGIILFLFLFSV ISFFVGKSMAEHDTSLPSVTTESFDGVQSSSGAHNILILGSDTRGEDAGRADTIMVLQLD GPAHKPKLISFMRDSFVAIPGVGQNKINAAYAYGGADLVRQTIKENFGLDCQYYAKVDFK SFEKVVDALFMNGIKIDAEKDLNLDGVDIKKGVQKMDGHTLLQYARFRKDEEGDFGRVRR QQQVMNTIFSQLKNPLNLIRAPYAAGKAIGYTSTDVSSFFIIKNLLSIARGVGGVDRLSV PVEGSWNFGNSSYAGSILVIDNDANRAAISDFLSK

> *Enterococcus hirae lcpC* gene sequence ATGAAGCGGTGGCAAAAAGTCGTAATAGCTTTACTGGGAATTCTCGTTGTTTTTATTGGC GGAGTCTCAGCTTATGGAATTAAACTCATGGGTGAAGCAAACCAAACAGTCAATCAAATA TCCAAGGGATCGAATCGTCAGTCAACAAAGCGTAAAGATAAAGTGAGTATTGACGACAAA GAACCTTTTTCTGTCCTTTTGTTAGGACTGGATACTGGCGGATTAGGTAGAACCGAACAA GGAAGATCGGATACAATGATGGTGGTAACGGTGAATCCCCAACAAAAAAAATCAACGATC GTCAGCTTAGACCGTGATATCTATACCAATATCGTTGGTCATGGTACTGTTGATAAGTTG AATCACGCCTATGCTTTTGGAGGCGTTGAAATGGCCATGGACTCAATCGAACAGTTACTC GATATCCCCATTGACCATTATGTAACCATTAATTTAGATGGAATGGAAGATTTGATCAAT GCTGTCGGAGGTGTGGAAGTCAATAATAAAATTGATTTTACATTAGATGGTGTTCATGTT CCTGTAGGTAAACAAGTATTAGATGGTAAGAAAGGCCTTGCTTATTCACGGATGCGTCAC GAAGATCCTAAGGGGGATATCGGACGTCAAGCACGACAGCGTGAAGTCGTTACCAAAATT GTTAATAAAGTATTGAGTTTAGATGGTGTCAGCAATTATCGGAAAATTTTAAAAGCGGTC GAAAAAAATGTCACAACTGACCTAGATTGGGATGATATGCTAGATGTAGCAACTAATTAC

ACACCAGCATTTGAAACGATCAAACAAGATCAATTGCAAGGTAAAGATGCAACCATTGAT AGTATTTACTATCAAATTTTAGGTGAAAATGATTTATTAAGTATCCAAAACGAATTGAAA AAACAATTAAACATCAAACCAAGTAAGACATTACCTAATTTAAAAAATGACAATGCTTCT ATCATGTTCTACAATGACTCGCAATCCGCTGAAGATTCGAACGAGACTGCGACAAGTGAG AATAATTCAACTTATTCAGGAAATTCGCAAACAACAAATCAGTACAACGAGTATAATCAA GAATACAATCAAAGCGCCGATCAAAATTATGATCAGTACGGCAATCAGAACTACAATCAG AATTACAACCAAAACAATGAACAAACTTACCAACAAGAAAACAATGAACAAGGATATAAT TATGGAAATGGATATTAA

> *Enterococcus hirae* LcpC amino acid sequence MKRWQKVVIALLGILVVFIGGVSAYGIKLMGEANQTVNQISKGSNRQSTKRKDKVSIDDK EPFSVLLLGLDTGGLGRTEQGRSDTMMVVTVNPQQKKSTIVSLDRDIYTNIVGHGTVDKL NHAYAFGGVEMAMDSIEQLLDIPIDHYVTINLDGMEDLINAVGGVEVNNKIDFTLDGVHV PVGKQVLDGKKGLAYSRMRHEDPKGDIGRQARQREVVTKIVNKVLSLDGVSNYRKILKAV EKNVTTDLDWDDMLDVATNYTPAFETIKQDQLQGKDATIDSIYYQILGENDLLSIQNELK KQLNIKPSKTLPNLKNDNASIMFYNDSQSAEDSNETATSENNSTYSGNSQTTNQYNEYNQ EYNQSADQNYDQYGNQNYNQNYNQNNEQTYQQENNEQGYNYGNGY

> *Bacillus subtilis* TagT (GenBank: AIC46295.1) MEERSQRRKKKRKLKKWVKVVAGLMAFLVIAAGSVGAYAFVKLNNASKEAHVSLARGEQSVK RIKEFDPGKDSFSVLLLGIDAREKNGETVDQARSDANVLVTFNRKEKTAKMLSIPRDAYVNI PGHGYDKFTHAHAYGGVDLTVKTVEEMLDIPVDYVVESNFTAFEDVVNELNGVKVTVKSDKV IQQIKKDTKGKVVLQKGTHTLDGEEALAYVRTRKADSDLLRGQRQMEVLSAIIDKSKSLSSI PAYDDIVDTMGQNLKMNLSLKDAIGLFPFITSLKSVESIQLTGYDYEPAGVYYFKLNQQKLQ EVKKELQNDLGV

> *Bacillus subtilis* TagU (GenBank: AIC46274.1) MRNERRKKKKTLLLTILTIIGLLVLGTGGYAYYLWHKAASTVASIHESIDKSKKRDKEVSIN KKDPFSVLIMGVDERDGDKGRADTLIYMTVNPKTNTTDMVSIPRDTYTKIIGKGTMDKINHS YAFGGTQMTVDTVENFLDVPVDYFVKVNMESFRDVVDTLGGITVNSTFAFSYDGYSFGKGEI TLNGKEALAYTRMRKEDPRGDFGRQDRQRQVIQGIINKGANISSITKFGDMFKVVENNVKTN LTFDNMWDIQSDYKGARKHIKQHELKGTGTKINGIYYYQADESALSDITKELKESLEK

> *Bacillus subtilis* TagV (GenBank: AIC46261.1) MAERVRVRVRKKKKSKRRKILKRIMLLFALALLVVVGLGGYKLYKTINAADESYDALSRGNK SNLRNEVVDMKKKPFSILFMGIEDYATKGQKGRSDSLIVVTLDPKNKTMKMLSIPRDTRVQL AGDTTGSKTKINAAYSKGGKDETVETVENFLQIPIDKYVTVDFDGFKDVINEVGGIDVDVPF DFDEKSDVDESKRIYFKKGEMHLNGEEALAYARMRKQDKRGDFGRNDRQKQILNALIDRMSS ASNIAKIDKIAEKASENVETNIRITEGLALQQIYSGFTSKKIDTLSITGSDLYLGPNNTYYF EPDATNLEKVRKTLQEHLDYTPDTSTGTSGTEDGTDSSSSSGSTGSTGTTTDGTTNGSSYSN DSSTSSNNSTTNSTTDSSY

> *Staphylococcus aureus* LcpA (NCBI Reference Sequence: YP_499888.1) MDKETNDNEYRRQSEHRTSAPKRKKKKKIRKLPIILLIVVILLIALVVYIVHSYNSGVEYAK KHAKDVKVHQFNGPVKNDGKISILVLGADKAQGGQSRTDSIMVVQYDFINKKMKMMSVMRDI YADIPGYGKHKINSAYALGGPELLRKTLDKNLGINPEYYAVVDFTGFEKMIDELMPEGVPIN VEKDMSKNIGVSLKKGNHRLNGKELLGYARFRHDPEGDFGRVRRQQQVMQTLKKEMVNFRTV VKLPKVAGILRGYVNTNIPDSGIFQTGLSFGIRGEKDVKSLTVPIKNSYEDVNTNTDGSALQ INKNTNKQAIKDFLDED

> *Staphylococcus aureus* LcpB (NCBI Reference Sequence: YP_499549.1) MNKFLKYFLILLALVLIVVPIVFATLLFKTSQDAFESSQDSKNANRQSNLRDNKVNPEEQPI SILFLGIDDNDGRRKKGQDAEHSRSDAMILTTFNQSKHQIRMLSIPRDTISYIPKVGYYDKI THAHAYGGPIAAMDSVEATMNVPVDYYVRVNMKAFVEAVNELGGIYYDVPYDLNEPNTDDTG KIKIKKGYQKLNGDEALAVARTRHHDSDLKRGQRQMELIKILFQKAQEVDSIDKLDNVIQIV GKNAKHNLTNSEIKALAKMYLTNDVEIKTAQLKGKDDMLNGIYYYHPSVESIQKYANLLRKD LELSPINDKNDFLDQRVINHYGSLIPLTPLDNSLLRKEQNDTTDKDKTSNENSDSTNNSDSS NQQQPATDQNSNQNQGGTQQAPQASNNQNGVVN

> *Staphylococcus aureus* LcpC (NCBI Reference Sequence: YP_501045.1) MSLPKKIFLWVFGILVILAIVAVVYVAAKIFITGNKIHNPLDRNHSELRDKKVSLNDGDPFT IALFGVDSDADRKKKGGGERSDSIMILSINPKTKKTEIVSIPRDTRAEIVGRGTTEKIAHAY AYGGPNMAVKSLEKLMNVPIDHYATIDMDGLHNMIDSIGGVDVVSNDTFTVDGVRFTKGQQT HVNGDQALKFIRSRKEEGAGGDFGRQQRQQIVLEAMANKIASPSSITHFNSLMNEIQNNVKT DLTLGDLNTIRSNYKDANDTINKHQLSGQGGIQSDGLYYFIPSEQSKAESTKLLKDNLE

From the multiple alignment presented from figure 1D the following pattern can be extracted R-X-D-X(20)-R-D-X(91,103)-R-X-R-X(4,7)-D-X(2)-R-X(2)-R-Q. The side-chains of these

residues replaced in the 3D structure of *Bacillus subtilis* TagT (YwtF, PDB accession number: 4DE9) show they are spatially close and some of them are involved in the binding of the pyro- phosphate lipid present into the TagT cavity.


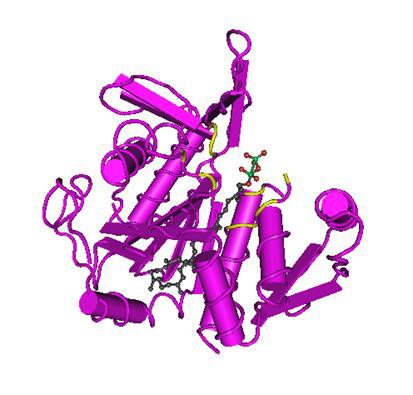


Structure (PDB 4DE9) of the complex between *Bacillus subtilis* TagT (YwtF) and octaprenyl- pyrophosphate. The conserved residues present in the pattern characteristic to LCP proteins are shown in yellow. The pyro-phosphate lipid is represented in “full atom”.

Amino-acid sequence of *B. subtilis* TagT:

>4DE9:A|PDBID|CHAIN|SEQUENCE

1 MASKEAHVSL ARGEQSVKRI KEFDPGKDSF SVLLLGIDAR EKNGETVDQA

51 RSDANVLVTF NRKEKTAKML SIPRDAYVNI PGHGYDKFTH AHAYGGVDLT

101 VKTVEEMLDI PVDYVVESNF TAFEDVVNEL NGVKVTVKSD KVIQQIKKDT

151 KGKVVLQKGT HTLDGEEALA YVRTRKADSD LLRGQRQMEV LSAIIDKSKS

201 LSSIPAYDDI VDTMGQNLKM NLSLKDAIGL FPFITSLKSV ESIQLTGYDY

251 EPAGVYYFKL NQQKLQEVKK ELQNDLGVLE HHHHHH

LcpA immunolocalization test in *E. hirae* R40 cells (*lcpA-*). No signal was observed for LcpA protein in this strain.


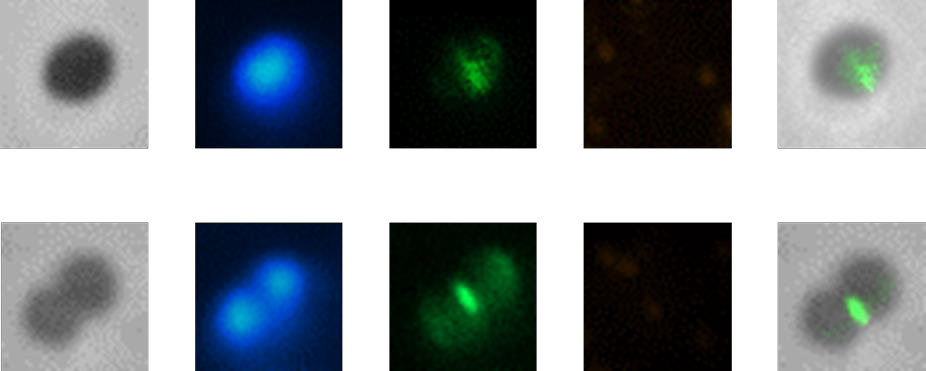


Cells were stained for DNA with DAPI (blue), peptidoglycan synthesis regions with fluoresce nt vancomycin (green), LcpA with anti-LcpA primary antibodies and Cy3-conjugated secondary antibodies (red). The upper panels show cells at an early division stage, as indicated by the presence of a single nucleoid and no apparent sign of midcell constriction. The lower panels show cells at a later stage of division, as indicated by the presence of a double nucleoid and a clear sign of midcell constriction.
